# Large carpenter bees show high dispersal in a tropical semi‐arid region susceptible to desertification

**DOI:** 10.1002/ece3.70085

**Published:** 2024-08-19

**Authors:** Sandara N. R. Brasil, Francisca Soares Araujo, Alan Brelsford, Christiana M. A. Faria, Lorenzo R. S. Zanette, S. Hollis Woodard

**Affiliations:** ^1^ Graduate Course of Ecology and Natural Resources, Department of Biology Federal University of Ceará Fortaleza‐CE Brazil; ^2^ Department of Evolution, Ecology, and Organismal Biology University of California, Riverside Riverside California USA; ^3^ Department of Entomology University of California, Riverside Riverside California USA

**Keywords:** Apoidea, ddRADseq, landscape, population genetic, population structure

## Abstract

Desertification is a major threat to biodiversity in arid areas of the world, partly because many organisms in these regions already exist at or near the limits of their movement and physiology. Here, we used molecular data to investigate patterns of persistence and dispersal in an ecologically and economically important carpenter bee (*Xylocopa grisescens* Lepeletier) found throughout the semiarid Caatinga region of Brazil. We used a genome‐wide approach (double digest restriction‐site associated DNA, ddRAD) to gather genetic data from bees sampled from eight sites within a semiarid region subject to desertification in Northeastern Brazil. Across all populations, we observed a consistent heterozygosity and effective population size deficit along with low genetic differentiation. We did not find strong evidence of dispersal limitations caused by desertification in this study system despite data collection from sites up to 300 km distant. Thus, our data suggest that human‐mediated changes in the Caatinga, such as habitat loss, have impacted the population genetic patterns of *X. grisescens*. However, these impacts have also been softened by the species' biological characteristics, such as its relatively high capacity for movement. This study provides insights into how habitat changes might impact the long‐term survival of large solitary bees.

## Graphical abstract

This study examines the genetic status of native carpenter bees in a Brazilian region vulnerable to desertification. Employing a population genomic approach, we found low genetic diversity with high homogeneity across populations, even over distances exceeding 300 km. The findings suggest that these bees may counteract desertification and habitat degradation through their mobility, facilitating gene flow and maintaining a continuous population distribution.

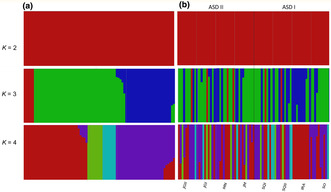

## 1 INTRODUCTION

Drylands, which collectively refer to arid, semiarid, and dry sub‐humid areas, represent 41% of the world's current land cover (Huang et al., 2017). An estimated 10%–20% of these areas are already severely degraded due to desertification, the progressive transformation of tropical forests in savannas and other dry ecosystems, highly driven by changes that include human‐mediated CO_2_ emissions and global warming (Verstraete, 1986; UNCCD, 1994; Millennium Ecosystem Assessment, 2005; Reynolds et al., 2007; Huang et al., 2017). Substantial species declines are already detected in drylands worldwide (e.g., Durant et al., 2014; Iknayan & Beissinger, 2018; Riddell et al., 2021; Williams et al., 1985). These declines are predicted to continue as aridity and habitat degradation continue to increase (Millennium Ecosystem Assessment, 2005). Hence, it has become increasingly important to understand and predict species responses to the expanding force of desertification as it continues to shape our planet and its biodiversity.

In arid landscapes, patterns of movement and dispersal are influenced by many interacting factors, including direct limits on physiological tolerances related to heat and water loss and dramatic inter‐annual differences in rainfall, which can alter dispersal‐related species interactions (Oliveira et al., 2019). Additionally, resources are often patchily distributed in arid landscapes (Reynolds et al., 2007), requiring animals to move between highly fragmented and often unrewarding habitats. These challenges are further exacerbated by human‐mediated land degradation, which reduces the total amount of available habitat and potentially increases habitat fragmentation by breaking apart the habitat and isolating suitable habitat patches (Galvin et al., 2008; Hadley & Betts, 2012; Haila, 2002; Rubinoff et al., 2020). Such limitations to dispersal can further result in limited gene flow between populations, causing high levels of genetic structure and population inbreeding, low effective population sizes, and, ultimately, local extinctions (Balkenhol et al., 2016; Davis et al., 2010; Templeton et al., 1990).

Habitat loss combined with fragmentation can also play a crucial role in shaping the evolution of dispersal traits. It can increase selection pressure for dispersal, as individuals may experience higher competition for limited resources within patches (Templeton et al., 1990). This selects traits that enhance the ability to disperse to new, less crowded patches where resources may be more abundant. In that case, dispersal can mitigate inbreeding by introducing genetic variation into isolated populations. Dispersal impacts on gene flow can be especially harmful to smaller‐bodied animals such as bees because their total amount of movement is limited by their relatively small size (Greenleaf et al., 2007). However, there is increasing evidence that bees are relatively mobile organisms (Ballare & Jha, 2020; Exeler et al., 2008; Jaffé et al., 2016; Suni, 2017), with traits such as body size and sociality appearing to mediate environmental effects on movement and genetic structure (Kendall et al., 2022; López‐Uribe et al., 2019). Moreover, some specific traits of desert‐dwelling bees, such as thermoregulatory capacity, time of emergence, and foraging strategies (Chappell, 1982; Danforth, 1999; Linsley, 1978; Minckley et al., 2000; Minckley & Ascher, 2012; Stone et al., 1999; Willmer & Stone, 1997), have allowed them to endure and even diversify in deserts and may allow them to persist in these regions into the future despite ongoing changes (Minckley & Radke, 2021; Orr et al., 2020). Understanding how desert habitats and ongoing desertification influence gene flow, genetic structure, and diversity in bees is critical to conserving this imperiled pollinator group. Yet, these patterns are still broadly not understood for an overwhelming majority of bee taxa (but see Danforth et al., 2003).

Large carpenter bees (genus *Xylocopa*) are globally distributed pollinators and include more than 730 species with essential roles in a wide range of crops and native plant species (Gerling et al., 1989; Keasar, 2010). The group's importance is partly due to their relatively large body size and floral sonication for pollen harvesting, which enhances their efficiency as pollinators and supports the reproduction of a wide variety of flowering plants (Buchmann, 1983; Gerling et al., 1989). Climate change and land degradation in the Neotropics, however, are predicted to cause a loss of ca. 35% to 47% of suitable habitats for *Xylocopa* species in the next 60 years (Bezerra et al., 2019), posing a significant threat to these carpenter bee species, the essential pollination services it provides and food security in the region (Klein et al., 2020). *Xylocopa grisescens* is a native species found throughout Brazil, particularly in the Brazilian Caatinga, a subtropical xeric forest and shrubland ecoregion covering much of the northeastern corner of the country (da Silva et al., 2018; Marchi & Alves‐dos‐Santos, 2013; Martins et al., 2014). The region has suffered from continuous human‐caused degradation due to extensive livestock farming and poor agricultural practices since European settlers' arrival, which accelerated following the 20th century (Kauffman et al., 1993; Veiga, 2007). As a result, the region has experienced increased environmental degradation, such as soil erosion and reduced natural regeneration capacity (Sampaio et al., 2017).

Here, we assess for the first time the population genomic status of a bee experiencing significant desertification in its habitat and across a large (~300 km^2^) portion of its range, which includes the higher‐risk areas of desertification in Brazil. Using a large number of markers obtained from ddRAD sequencing, we investigate the effects of habitat degradation and increased aridity on gene flow, genetic diversity, population structure, and the effective population size of a wild bee. Specifically, we test the predictions that *X. grisescens* populations would (1) exhibit low levels of heterozygosity due to decreased gene flow and prolonged isolation, in particular between areas susceptible to desertification; (2) exhibit significant genetic differentiation and be highly structured (non‐panmictic), reflecting inbreeding and dispersal limitations; and (3) show low effective population size in response to dispersal limitations.

## 2 MATERIALS AND METHODS

### 2.1 Study species

*Xylocopa grisescens* is a large carpenter bee easily recognized by its hairy body and dense white hairs in the mesosoma. It is solitary and exhibits high nest‐site philopatry (Klein et al., 2020; Marchi & Alves‐dos‐Santos, 2013). Nests are usually excavated in standing or fallen dead wood, but poles and construction wood can also be used (Klein et al., 2020; Martins et al., 2014; Neves et al., 2006). *X. grisescens* is an important generalist native pollinator found throughout Brazil, particularly in the semiarid region (Marchi & Alves‐dos‐Santos, 2013; Neves et al., 2006). The economic importance of the species relies on its effective pollination of many crop plant species, especially in the passion fruit family (*Passiflora edulis* Sims f. *flavicarpa*, Junqueira & Augusto, 2017; Siqueira et al., 2009), of which Brazil is the world's largest producer (USAID, 2014).

### 2.2 Sampling and study area

**FIGURE 1:**
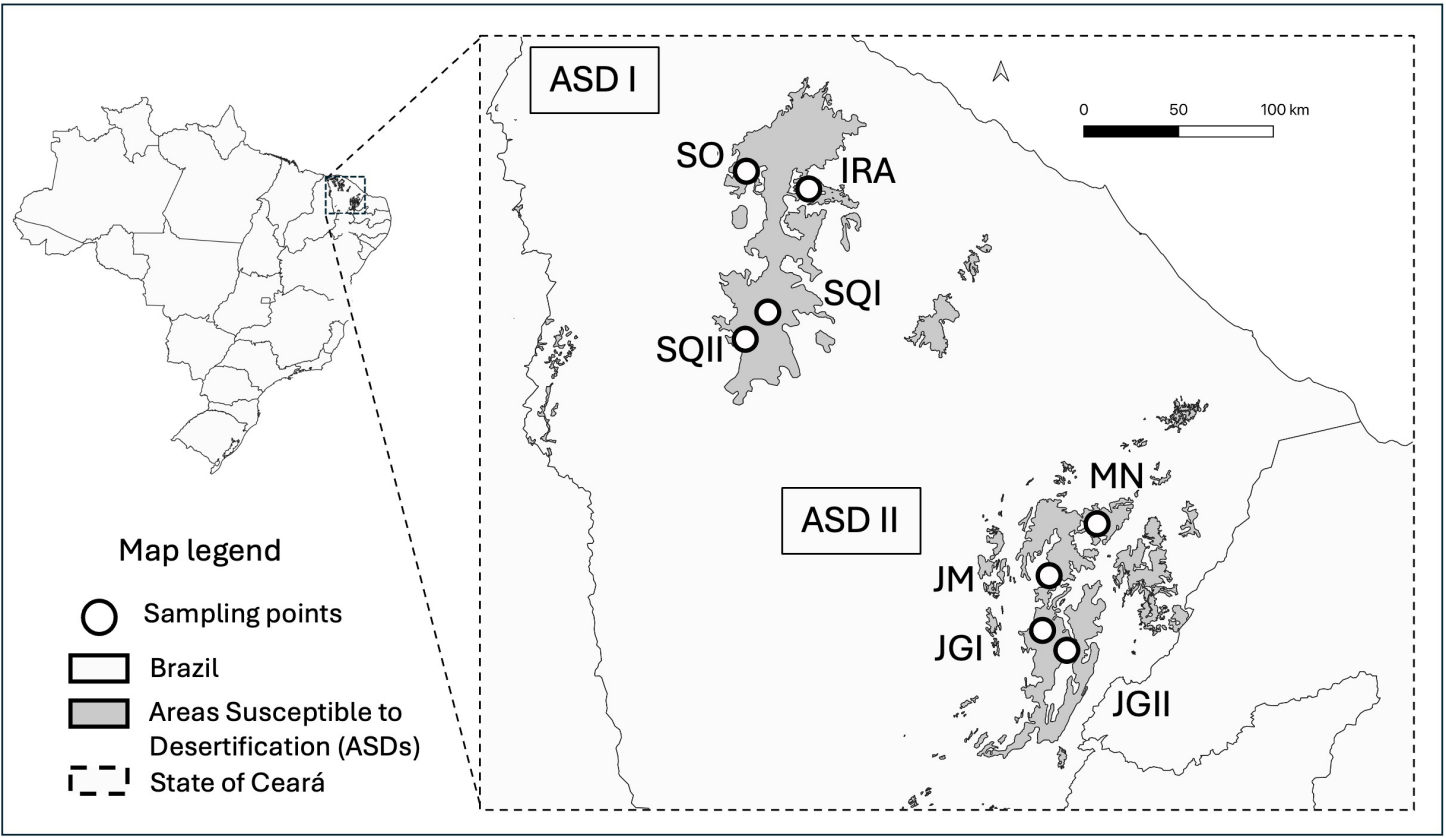
Map of geographic sampling locations of *X. grisescens*. The location of Brazilian states within the country's territory, as well as details of the State of Ceará delimitation (Brazilian northeast) and its respective areas susceptible to desertification ASDI and ASDII. White dots represent the sampling points of the eight populations, with names (acronyms) provided in Table 1 and Table S1.

Bees were sampled in eight sites throughout the State of Ceará in Northeast Brazil, clustered in two distinct areas susceptible to desertification (hereafter called ASDs; Figure 1). Northeastern Brazil is classified as dryland, and it is one of the world's most climate‐vulnerable regions (IPCC, 2013). The predominant vegetation in the region, locally known as Caatinga, is characterized by a primarily deciduous xerophilous thorn woodland/shrubland with ephemeral seasonal herbaceous stratum, mainly composed of therophytes (da Costa et al., 2007). Since Brazilian colonization in the sixteenth century, the Caatinga ecoregion has declined from an area of ~900,000 km^2^ to remnants that correspond to only ~40% of the original cover (da Silva et al., 2018). In addition, cattle overgrazing, slash‐and‐burn agriculture, replacement of native vegetation by crops, and natural wood removal for charcoal production have established a continuum of degradation that has dramatically increased the rate and risk of desertification in the Caatinga areas domain (Sampaio et al., 2017).

From each site, ten female *X. grisescens* were collected using sweep nets while foraging on flowers and immediately preserved in 95% ethanol. Samples were subsequently stored at −20°C. Geolocation data for sampling points are provided in Table S1 (Appendix S1). Sampling sites were at least 16 km apart to minimize the correlation between sites, considering that some species of *Xylocopa* can typically fly up to 1.2 km when foraging (Greenleaf et al., 2007).

### 2.3 DNA extraction and library preparation

DNA was extracted from one middle leg of each individual using a Qiagen DNeasy Blood and Tissue kit (Qiagen, 2006), following the manufacturer's instructions with minor modifications. We used 70% ethanol instead of AW2 and 30 μL of AE elution buffer. DNA quality and concentration were verified using a Qubit 3.0 (Life Technologies). We generated double digest restriction site‐associated DNA (ddRADseq) libraries for all 80 bees using the method of Brelsford et al. (2016), which incorporates elements of those proposed by Parchman et al. (2012) and Peterson et al. (2012), Briefly, genomic DNA was digested using *Mse*I and *EcoR*I restriction enzymes (Illumina, Inc.), followed by the annealing of two unique adapters for each DNA fragment (forward and reverse). Ligated samples were purified with Agencourt AMPure beads (Beckman Coulter, Inc.) to remove small DNA fragments, and then two PCR amplifications were performed. All libraries were multiplexed in a single lane and sequenced on an Illumina HiSeq X‐Ten paired‐end 150 bp platform at Novogene Corporation (https://en.novogene.com).

### 2.4 Bioinformatic data processing

Following this, we used the *process_radtags* tool in Stacks v2.3b (Catchen et al., 2011, 2013) with default parameters to demultiplex raw data by barcode, remove any read with an uncalled base (*−c*), discard reads with low‐quality scores *(−q*), and rescue barcodes and RAD‐Tag cut sites (*−r*). We then ran the *denovo_map* pipeline to perform a de novo assembly of loci for all populations, allowing only three mismatches within individuals (−M 3) and four between individuals (−n 4), and allowed loci present in at least 80% of individuals (*−r 80*). This resulted in 42,729 SNPs and a mean loci length of 300 bp. The resulting VCF file was filtered to include only high‐quality SNPs with a quality score > 20, and only SNPs successfully genotyped in 60% of the individuals. To ensure we removed any possible paralogs, we also used the flag ‐‐hardy to check for heterozygosity excess (*p*‐value < .05) and excluded all sites with excess heterozygosity using the flag ‐‐exclude‐positions. Next, we filtered the dataset to include SNPs with a minimum mean depth of 10x (‐‐min‐meanDP 10). VCFtools was also used to convert VCF files to different file formats and to assess locus and sample coverage depth statistics.

### 2.5 Population genetic analysis and inference of genetic structure

We used the *populations* module in Stacks to calculate expected heterozygosity (*H*_e_), observed heterozygosity (*H*_o_), private allele number (*A*_p_), percentage of polymorphic loci (%*P*), and inbreeding coefficient (*F*_*IS*_) for each population. We set the minor allele frequency (*‐‐min‐maf*) to <0.05 and used the flag *‐‐write‐single‐SNP*, which keeps only one SNP per locus to reduce the effects of linkage disequilibrium. To investigate the genetic structure, we used three main approaches. First, we calculated pairwise SNP‐related haplotype‐based F statistics (*F*_*ST*_) using the *populations* program in Stacks v2.3. Second, we used PLINK v.1.9 (Purcell et al., 2007) to perform a Principal Component Analysis (PCA) by extracting PC coordinates for each individual. Lastly, we generated bed, bim, and fam files in PLINK to be analyzed on the Bayesian model‐based clustering program fastStructure (Raj et al., 2014). We evaluated prior clusters from *K* = 1 to *K* = 8, with 20 independent runs for each *K* (where *K* equals the population clusters) to map genetic structure and common kinship. The optimal value of *K* was obtained by running the flag c*hooseK.py* according to two metrics to obtain a range of values for the number of populations that explain the structure in data: (1) model complexity that maximizes the marginal likelihood of the entire data, and (2) model components used to explain structure in data. We further visualized the observed admixture for chosen values of *K* using the flag *distruct.py*. All plots were created using the R packages ggplot2 v.3.3.2 and tidyverse v.1.3.0 (Wickham et al., 2019, 2020). We also investigated genetic relatedness in our dataset using the VCFTools ‐‐relatedness parameter (Table S2). To estimate the effective population size for our eight populations, we used a linkage disequilibrium‐based method as carried out in NeEstimator v2.1 with default parameters (Do et al., 2014).

### 2.6 Isolation by distance

To test Isolation by distance (IBD), we constructed two distance matrices (genetic distance and geographic distance) and tested their correlation using MRDM (multiple regression on distance matrices) in the R package ecodist (Goslee & Urban, 2007) with 10,000 permutations. The matrix of genetic distances between populations was constructed by calculating pairwise *F*_*ST*_ using VCFtools (Danecek et al., 2011) and set as the response variable, while the geographic distance matrix was calculated from the sample site coordinates using the function vegdist in the vegan package (Oksanen et al., 2015) and set as the explanatory variable. We plotted pairwise *F*_*ST*_ against geographic distances as a scatter plot using the package ggplot2 v.3.3.2 in R to better visualize this correlation.

## 3 RESULTS

### 3.1 SNP calling and filtering

RAD sequencing resulted in ~917 million raw reads and ~ 815 million retained high‐quality reads with a mean effective coverage per individual of ~36x (7.5x–81.6x). The de novo assembly generated a total of ~1,370,000 loci with a mean length of 300 bp for the entire dataset. Final dataset resulted in 6397 single nucleotide polymorphisms (SNPs). The frequency of missing data ranged from 0.1 to 0.7, with a mean of 0.20 (±0.1). Additional information on missing data for the dataset can be found in Table S1 (Appendix S1).

### 3.2 Population genetic diversity

We observed a low variance in genetic diversity among all localities (Table 1). No significant difference was found among all sites when analyzing the percentage of polymorphic loci (%*P*) (*p* = .248). This ratio, which was measured by dividing the number of polymorphic loci by the total number of loci, ranged narrowly from 7.8% (IRA) to 12.5% (JGI), denoting low variation among sites (4.7%) (Table 1).

**TABLE 1:** Summary statistics of estimates of genetics parameters based on 6397 SNPs from all 80 individuals of *X. grisescens*. Sample size of populations consisted of 10 individuals.

Population	NPa	*H*_O_	*H*_E_	*P* (%)	*F*_IS_	*N*_e_
SQI	245	0.139	0.184	0.106	0.209	7.3
IRA	104	0.113	0.166	0.078	0.235	2.9
SO	274	0.126	0.176	0.099	0.222	7.2
SQII	493	0.134	0.190	0.117	0.247	102.9
JGI	509	0.136	0.195	0.125	0.249	52.4
JM	346	0.122	0.186	0.106	0.269	15.5
JGII	261	0.131	0.180	0.101	0.221	10.5
MN	511	0.134	0.192	0.121	0.252	41.3

Abbreviations: *F*_IS_, inbreeding coefficient; *H*_E_, expected heterozygosity; *H*_O_, observed heterozygosity; *N*_e_, Effective population size; NPa, Number of private alleles; *P*(%), percentage of polymorphic loci.

Observed heterozygosity under Hardy–Weinberg Equilibrium (*H*_O_) significantly underperformed expected heterozygosity (*H*_E_) over localities (*t* = 12.094, df = 13.903, *p*‐value <.001). *H*_O_ ranged from 0.113 (IRA) to 0.139 (SQI), and similarly, *H*_E_ ranged from 0.166 (IRA) to 0.195 (JGI) (Table 1). Low variations in *F*_IS_ values were found for all localities ($\bar{x}$ = 0.238) (Table 1). Among sites, SQI showed the lowest value (*F*_IS_ = 0.209) followed by a slightly higher value in JGII (*F*_IS_ = 0.221), whereas MN and JM showed the highest mean values (*F*_IS_ = 0.252 and *F*_IS_ = 0.269, respectively) (Table 1). The number of private alleles (NPa) varied from 104 (IRA) to 511 (MN). Effective population size ranged from 2.9 in IRA and 102.9 in SQII (Table 1).

### 3.3 Population genetic structure and isolation by distance

**FIGURE 2:**
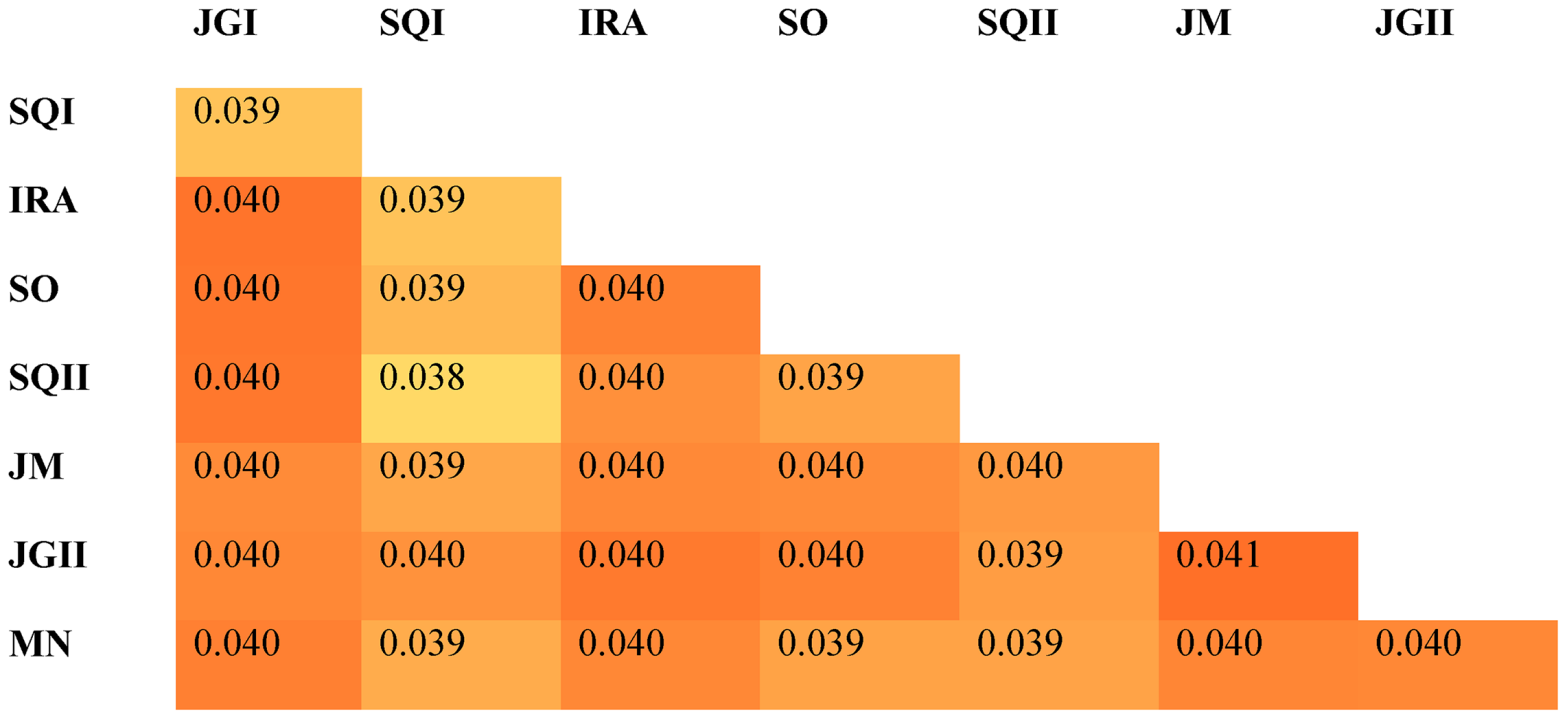
*F*_ST_ matrix calculated for each pair of populations. Values from each pairwise analysis are displayed inside the grid cells. Heatmap colors represent the strength of each correlation, whereas colors close to yellow represent a weaker correlation, and colors close to red represent a stronger correlation. The differences are not statistically significant.

**FIGURE 3:**
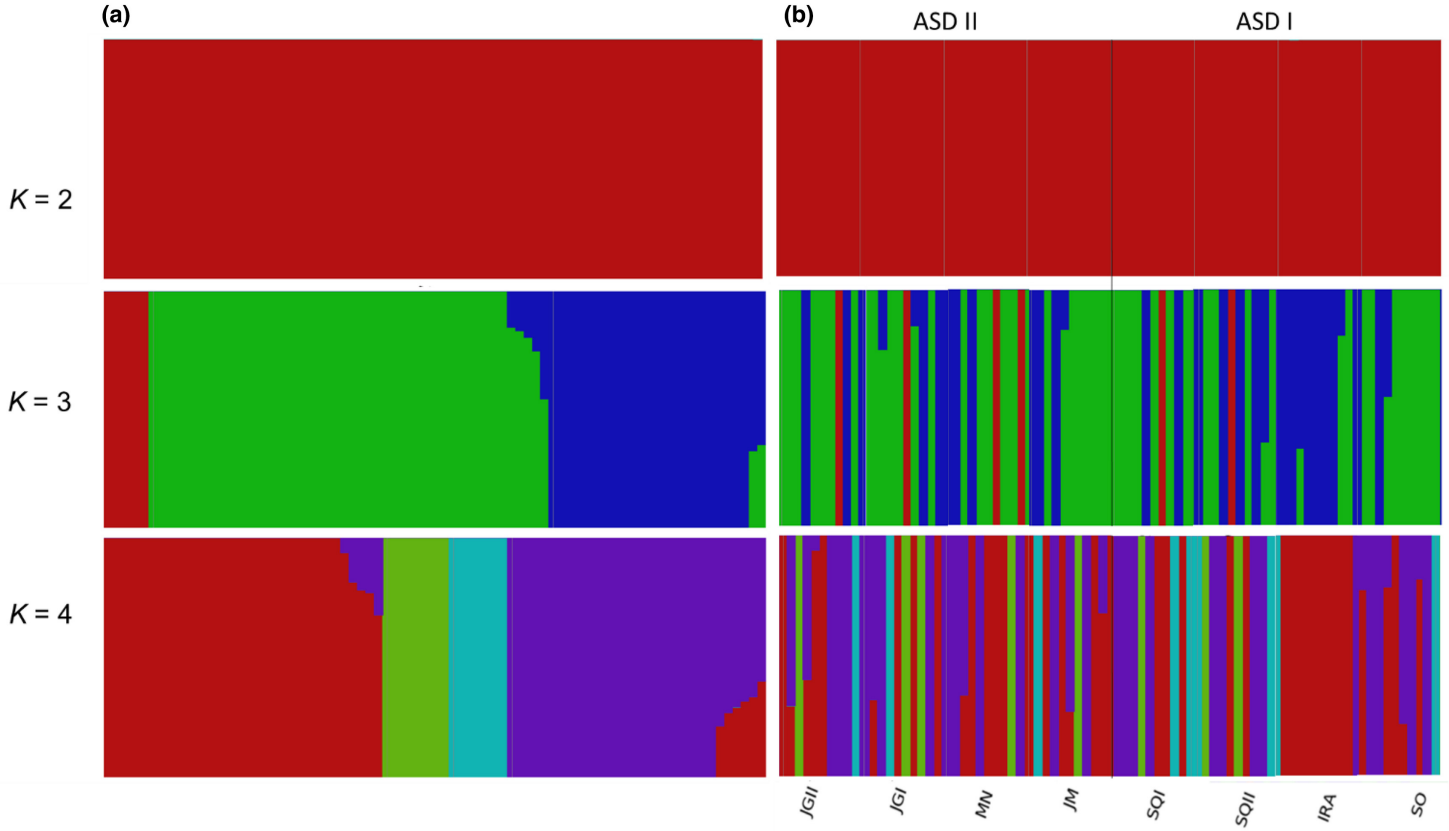
Population structure results obtained from fastSTRUCTURE analysis for eight sites based on genetic distance. Each vertical bar represents a single individual of *X. grisescens*, and colors represent unique ancestry proportions (membership) from *K* = 2 to *K* = 4 (marginal likelihoods are provided in Table S3, Appendix S1). (a) admixture proportions as inferred with no prior population designation; (b) admixture proportions according to each geographic location, including ASDs and sample sites. The black line separates ASD II and ASD I sites.

**FIGURE 4:**
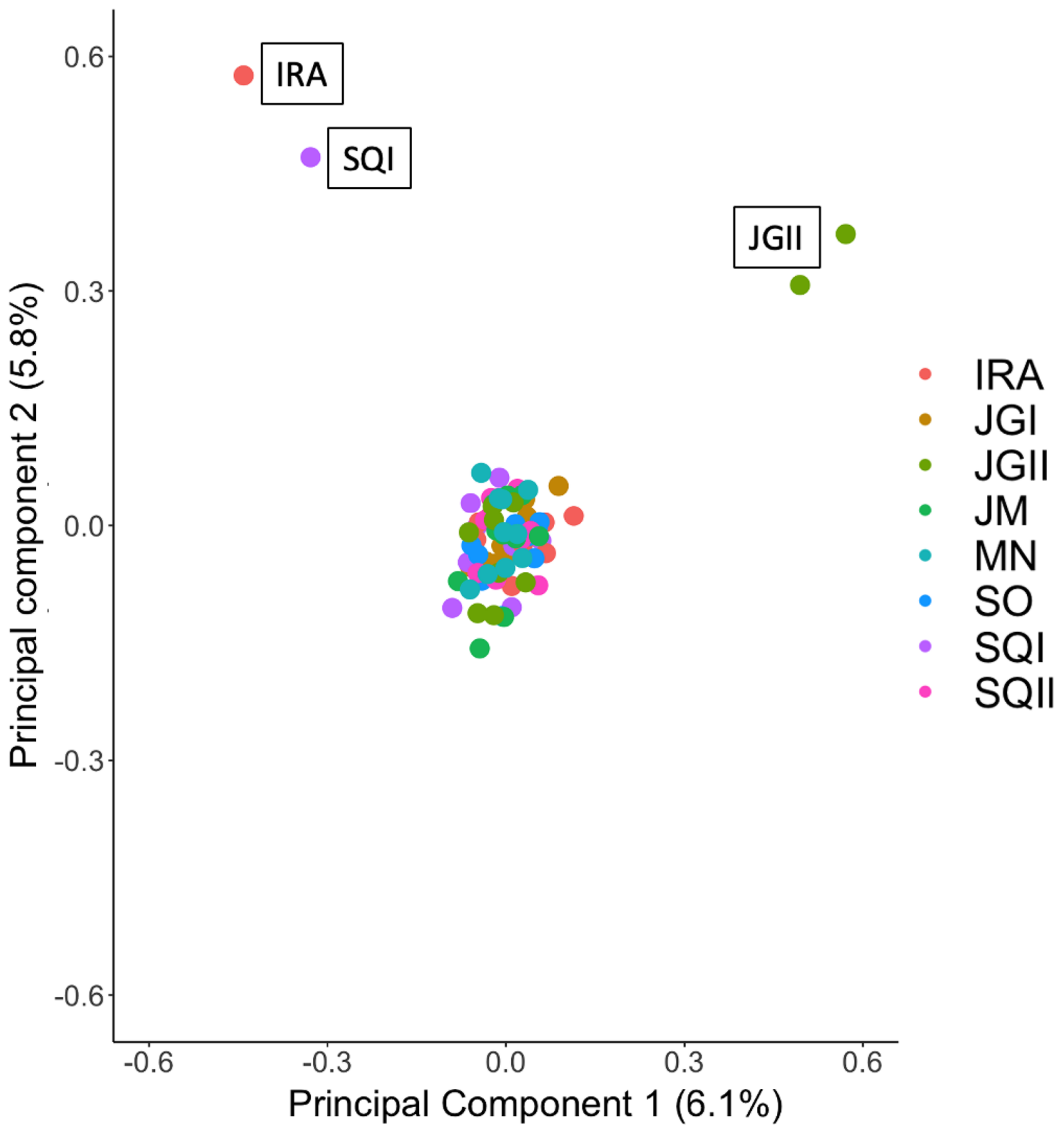
Population structure of *X. grisescens* revealed by PCA analysis based on the filtered dataset of 6397 SNPs. Scatter plot of PCA revealing one large cluster and two pairs of genetically similar inidviduals are represented as colored dots, whereas each color indicates a specific sample site.

The genetic differentiation between sites (*F*_ST_) ranged narrowly from 0.038 to 0.041, with the lowest value found between two very close sites, SQI and SQII (*F*_ST_ = 0.038), and the most substantial divergence was found between JM and JGII (*F*_ST_ = 0.041), which are >70 km apart (Table 1, Figure 2). PCA and fastStructure results revealed some genetic structure. Although not consistent with geographic groupings, a few dominant ancestry components are present in the admixture distributions, displayed by the different colors in Figure 3. Accordingly, the results from fastStructure suggested the optimal value of *K* to be *K* = 1 for the model complexity that maximizes marginal likelihood and *K* = 3 for the model used to explain structure in the data, with marginal likelihood values decreased when raising *K* (Table S3). Here, we considered the best *K* as having the highest marginal likelihood (*K* = 1). In the PCA plot, two genetically similar individuals from the same site (JGII) were far apart from the group cluster along the first PC axis. A second pair of genetically similar individuals (Ajk index of 0.33 from a global range of 0.0001 to 2.11, Table S2) from different sample sites were independently grouped along the second PC axis (Figure 4a).

Genetic differentiation expressed by F_ST_ did not increase with geographic distance across all pairwise comparisons. The multiple regression on distance matrices (RMDM) results of IBD showed low values of correlation (*R* ^2^ = 0.0038, *F*‐value = 0.0719), and no statistical significance (*p*‐value = .7021). Plotted values are provided in Figure S1.

## 4 DISCUSSION

We examined genome‐wide data from a wild bee (*X. grisescens*) to evaluate patterns of genetic diversity, structure, and differentiation in a dryland habitat subject to intensive and ongoing desertification. Our results indicate that mobility and gene flow in our focal species are quite high across the study area, even encompassing sites 300 km apart and spanning a matrix that includes areas undergoing considerable degradation. Overall, there is a trend of heterozygosity deficit. However, the genetic variation was consistently low, and the genetic structure showed no association with geographic distance. This is reflected in our findings, which show that only 0.38% of genetic differentiation can be explained by geographic distance and that the association between differentiation and distance is not statistically significant, reinforcing that no evidence of isolation by distance is detected in our data. Although previous studies have explored genetic variability and isolation in desert and semidesert taxa (e.g., plants, mammals, and reptiles; el Mousadik & Petit, 1996; Epps et al., 2005; Shaffer et al., 2017), our study is the first to use many thousands of neutral genetic markers to examine fine‐scale patterns of the genetic status of bee species facing desertification.

Human‐mediated habitat degradation can contribute to the loss of genetic variation (Balkenhol et al., 2016), ultimately driving species loss, and can be especially harmful to species in drylands because food resources and nest sites are often already sparsely distributed in these areas (Galvin et al., 2008). However, organisms can prevail over these effects if they move readily between habitat patches. Our results indicate that the latter may be the case for *X. grisescens*. Given the distances that we detected evidence of gene flow across (up to 300 km), it appears that this species has the capacity to migrate across large areas of the landscape. Moreover, the detection of two related individuals collected in sites 70 km apart suggests that individual can navigate highly fragmented landscapes, including across areas severely impacted by desertification. Our findings are consistent with other studies on non‐desert‐dwelling bees, which have found that despite their relatively small body size, bees are highly mobile and able to maintain movement and ongoing gene flow. For example, gene flow occurring over remarkably long distances (up to 200 km) has been reported in populations of the tropical stingless bee species *Trigona spinipes*, even across agricultural landscape mosaics and human‐altered forest fragments in Brazil (Jaffé et al., 2016). Likewise, Suni (2017) found evidence of weak population differentiation (suggesting unrestricted dispersal) for the orchid bee *Euglossa imperialis* over degraded landscapes in Costa Rica. Similar results of low population differentiation were found for the large carpenter bee *Xylocopa virginica* dwelling in human‐altered habitats (Ballare & Jha, 2020). Thus, there is an indication that wild bees are capable of maintaining genetic connectivity despite habitat fragmentation, suggesting that they possess traits that facilitate dispersal and adaptability in altered environments.

Our results show that *X. grisescens* sampled across a vast area at high risk of becoming a desert belong to a single genetic cluster. Similar findings of low genetic structure over large areas have been previously reported for wild bees, such as *Agapostemon virescens* and *Bombus terricola* (Kent et al., 2018; Samad‐Zada & Rehan, 2023). The large body size of *X. grisescens* might make it especially mobile and able to traverse great distances without being limited even by extended areas of degraded areas. Larger body sizes enable stronger flight muscles, thus permitting greater long‐distance foraging flight (Somanathan et al., 2019). Previous studies have indicated that as body size increases, species tend to expand their foraging ranges and, consequently, their habitat use (Kendall et al., 2022). Further, carpenter bees tend to be speciose in deserts (Michener, 1979; Orr et al., 2020) and have physiological adaptations that allow efficient water balance in arid environments and to remain active in desert regions even during the hottest parts of the day (Chappell, 1982; Nicolson & Louw, 1982; Willmer, 1988). Another carpenter bee (*Xylocopa frontalis*) in Brazil has been reported to maintain its body temperature above the ambient temperature during the hottest times of the day, reaching almost 47°C (de Farias‐Silva & Freitas, 2021). Such adaptations to hot environments might also facilitate movement and persistence in this bee group despite ongoing and even increasing aridity. Patterns of movement in *X. grisescens* may also be facilitated by features of the Caatinga landscape that allow bees to move more readily than predicted based on the typical patchiness of desert habitats or might even require them to be more mobile to persist. For the former, functional connectivity between habitat fragments might be eased by remnants of large‐sized vegetation patches across parts of the ecoregion (Antongiovanni et al., 2018). For the latter, given that food availability in the Caatinga ecoregion is strongly driven by short rainfall pulses that occur only a few months a year, this might require *X. grisescens* to track specific food resources across the landscape, as has been reported for bee species in arid regions (Minckley et al., 2013) and in temperate‐dwelling bumble bees (Jha & Kremen, 2013; Pope & Jha, 2018; Westphal et al., 2006).

Resource tracking of highly dispersed food sources for bees, i.e., single trees or small patches scattered across the Caatinga dryland, could potentially select for higher dispersing capacity in this bee. Regarding nesting opportunities, given that *X. grisescens* nests in natural substrates such as tree trunks and driftwood logs that are continuously available in the Caatinga region (Camillo & Garófalo, 1982; Chaves‐Alves et al., 2011), nesting substrates may be less of a limiting factor underlying the genetic patterns we observed, compared to the seasonal scarcity of food resources. Our results suggest that connectivity is maintained, but we cannot conclude whether genetic diversity has remained stable or changed recently. It is also possible that despite frequent and prolonged disturbances in the study area, which are assumed to negatively impact population genetic structure by limiting dispersal, the fragmentation effects may have occurred recently and may not yet be detectable at the genetic level. Our observation of the easy navigation of our focal bee in the Caatinga, overcoming potential barriers to dispersal, could also be explained by a recent study in the same areas affected by desertification. Macêdo et al. (2024) found that, within these ASDs, even severely degraded fragments still have a high biological potential for regeneration because remnants can act as reservoirs, maintaining regional biodiversity in the area.

Overall, our study sheds light on the genomic status of an important carpenter bee species in a degraded semiarid region. We suggest that for *X. grisescens*, under the conditions and scale we explored, gene flow is thus far maintaining populations with individuals resistant to geographic isolation and ongoing desertification. This maintenance of gene flow appears to be partially facilitated by this species' high mobility. Nevertheless, populations of this species have shown low effective population sizes across all sampled sites, which might be linked to the intensification of anthropogenic land use in the region. Future studies with *X. grisescens* and desert‐dwelling bee species will be critical for identifying how genetic diversity and structure patterns have shaped bee biodiversity in arid environments and their fates in response to increasingly human‐altered arid landscapes.

## AUTHOR CONTRIBUTIONS

**Sandara N. R. Brasil:** Conceptualization (equal); data curation (lead); formal analysis (lead); visualization (lead); writing – original draft (lead). **Francisca Soares Araujo:** Conceptualization (equal); funding acquisition (equal); project administration (equal); writing – review and editing (equal). **Alan Brelsford:** Formal analysis (supporting); methodology (equal); validation (equal); writing – review and editing (equal). **Christiana M. A. Faria:** Investigation (equal); methodology (equal); writing – review and editing (equal). **Lorenzo R. S. Zanette:** Conceptualization (equal); methodology (equal); project administration (equal); writing – review and editing (equal). **S. Hollis Woodard:** Data curation (equal); funding acquisition (equal); methodology (equal); supervision (equal); validation (equal); writing – original draft (equal); writing – review and editing (equal).

## CONFLICT OF INTEREST STATEMENT

We declare that there are no conflicts of interest.

## Supporting information


Data S1.

Appendix S1.

## Data Availability

Data for this study are available at Dryad under the following link: https://datadryad.org/stash/share/decFj9rWvlJhEjEmmc8hJkrAWF1B7hOAI7osLX‐JV70.
