# Supplementary material for: Large carpenter bees show high dispersal in a tropical semi‐arid region susceptible to desertification
Source: Ecol Evol. 2024 Aug 19;14(8):e70085. doi: 10.1002/ece3.70085 (PMC11333300; doi:10.1002/ece3.70085)
Supplement: Supplementary file 1 — Data S1. [file ECE3-14-e70085-s002.docx]

APPENDIX


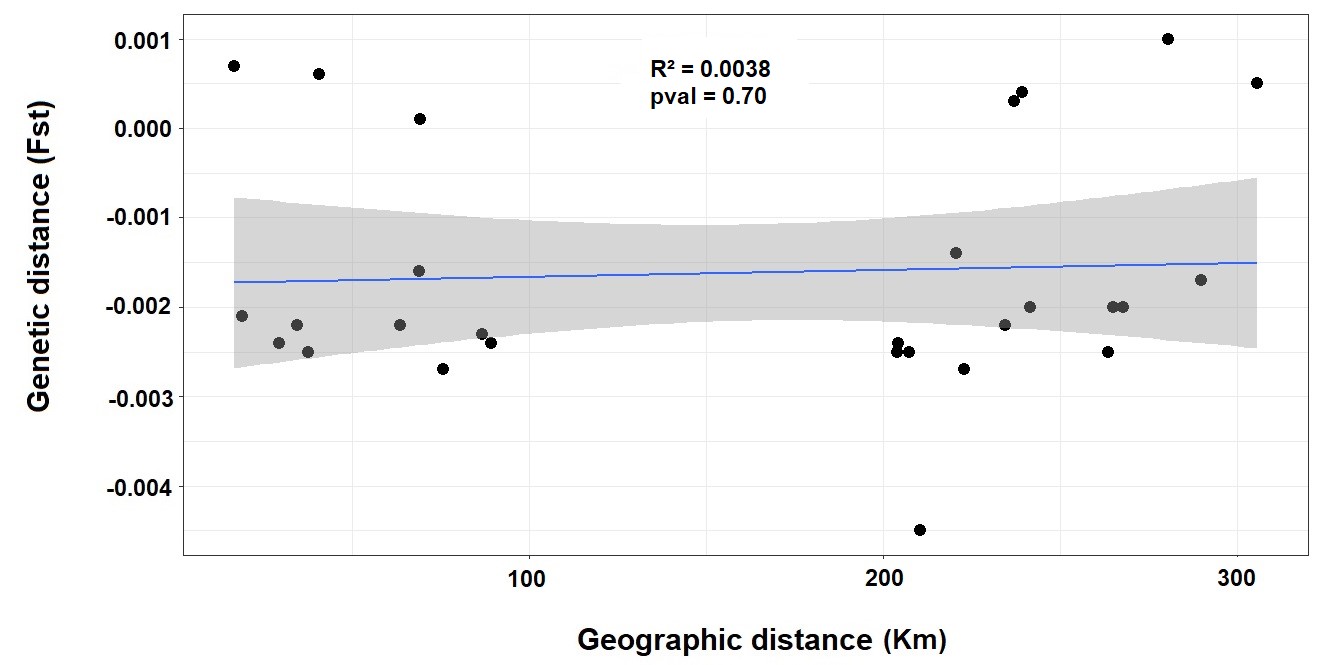


Figure S1. Scatter plot of MRDM analysis for isolation by distance showing the pairwise relationship between genetic distances and geographic locations of populations of *X. grisescens*. Genetic distances were obtained by calculating pairwise F_ST._ Geographic distances are displayed in kilometres (km). The test revealed no significant correlation (R² = 0.0038, pvalue= 0.7021) between analyzed matrices. The regression line is shown with a 95% confidence shadow.

Table S1. The number of variant sites, variant sites per sample and frequency of missing sites before quality filtering and after quality filtering for each sample site and locality.

|  |  |  |  | **Before quality filtering** | | | | **After quality filtering** | | |
| --- | --- | --- | --- | --- | --- | --- | --- | --- | --- | --- |
| **Sample ID** | **Locality name** | **Longitude** | **Latitude** | **number of sites** | **variant sites per sample** | **missing sites** | **frequency of missing** | **number of sites** | **missing sites** | **frequency of missing** |
| JG101 | Jaguaribe | -38.77 | -5.83 | 42729 | 21941 | 20788 | 0.4865 | 6397 | 927 | 0.144912 |
| JG102 | Jaguaribe | -38.77 | -5.83 | 42729 | 23054 | 19675 | 0.4605 | 6397 | 815 | 0.127403 |
| JG103 | Jaguaribe | -38.77 | -5.83 | 42729 | 22524 | 20205 | 0.4729 | 6397 | 879 | 0.137408 |
| JG104 | Jaguaribe | -38.77 | -5.83 | 42729 | 22741 | 19988 | 0.4678 | 6397 | 934 | 0.146006 |
| JG105 | Jaguaribe | -38.77 | -5.83 | 42729 | 23324 | 19405 | 0.4541 | 6397 | 721 | 0.112709 |
| JG106 | Jaguaribe | -38.77 | -5.83 | 42729 | 23214 | 19515 | 0.4567 | 6397 | 725 | 0.113334 |
| JG107 | Jaguaribe | -38.77 | -5.83 | 42729 | 23207 | 19522 | 0.4569 | 6397 | 792 | 0.123808 |
| JG108 | Jaguaribe | -38.77 | -5.83 | 42729 | 22823 | 19906 | 0.4659 | 6397 | 805 | 0.12584 |
| JG109 | Jaguaribe | -38.77 | -5.83 | 42729 | 22651 | 20078 | 0.4699 | 6397 | 923 | 0.144286 |
| JG110 | Jaguaribe | -38.77 | -5.83 | 42729 | 19696 | 23033 | 0.539 | 6397 | 1580 | 0.246991 |
| SQ101 | Santa Quitéria | -40.08 | -4.31 | 42729 | 17470 | 25259 | 0.5911 | 6397 | 1552 | 0.242614 |
| SQ102 | Santa Quitéria | -40.08 | -4.31 | 42729 | 18207 | 24522 | 0.5739 | 6397 | 1138 | 0.177896 |
| SQ103 | Santa Quitéria | -40.08 | -4.31 | 42729 | 18216 | 24513 | 0.5737 | 6397 | 1203 | 0.188057 |
| SQ104 | Santa Quitéria | -40.08 | -4.31 | 42729 | 17595 | 25134 | 0.5882 | 6397 | 1496 | 0.23386 |
| SQ105 | Santa Quitéria | -40.08 | -4.31 | 42729 | 18149 | 24580 | 0.5753 | 6397 | 1274 | 0.199156 |
| SQ106 | Santa Quitéria | -40.08 | -4.31 | 42729 | 18035 | 24694 | 0.5779 | 6397 | 1284 | 0.200719 |
| SQ107 | Santa Quitéria | -40.08 | -4.31 | 42729 | 12057 | 30672 | 0.7178 | 6397 | 3649 | 0.570424 |
| SQ108 | Santa Quitéria | -40.08 | -4.31 | 42729 | 17353 | 25376 | 0.5939 | 6397 | 1609 | 0.251524 |
| SQ109 | Santa Quitéria | -40.08 | -4.31 | 42729 | 16215 | 26514 | 0.6205 | 6397 | 1983 | 0.309989 |
| SQ110 | Santa Quitéria | -40.08 | -4.31 | 42729 | 17880 | 24849 | 0.5815 | 6397 | 1269 | 0.198374 |
| IRA01 | Irauçuba | -39.88 | -3.73 | 42729 | 13449 | 29280 | 0.6852 | 6397 | 1537 | 0.240269 |
| IRA02 | Irauçuba | -39.88 | -3.73 | 42729 | 5874 | 36855 | 0.8625 | 6397 | 4977 | 0.778021 |
| IRA03 | Irauçuba | -39.88 | -3.73 | 42729 | 13340 | 29389 | 0.6878 | 6397 | 1721 | 0.269032 |
| IRA04 | Irauçuba | -39.88 | -3.73 | 42729 | 13540 | 29189 | 0.6831 | 6397 | 1454 | 0.227294 |
| IRA05 | Irauçuba | -39.88 | -3.73 | 42729 | 13584 | 29145 | 0.6821 | 6397 | 1527 | 0.238706 |
| IRA06 | Irauçuba | -39.88 | -3.73 | 42729 | 13459 | 29270 | 0.685 | 6397 | 1525 | 0.238393 |
| IRA07 | Irauçuba | -39.88 | -3.73 | 42729 | 11889 | 30840 | 0.7218 | 6397 | 2619 | 0.409411 |
| IRA08 | Irauçuba | -39.88 | -3.73 | 42729 | 13431 | 29298 | 0.6857 | 6397 | 1748 | 0.273253 |
| IRA09 | Irauçuba | -39.88 | -3.73 | 42729 | 13409 | 29320 | 0.6862 | 6397 | 1517 | 0.237142 |
| IRA10 | Irauçuba | -39.88 | -3.73 | 42729 | 12555 | 30174 | 0.7062 | 6397 | 2236 | 0.349539 |
| SO01 | Sobral | -40.18 | -3.64 | 42729 | 11765 | 30964 | 0.7247 | 6397 | 3828 | 0.598406 |
| SO02 | Sobral | -40.18 | -3.64 | 42729 | 17154 | 25575 | 0.5985 | 6397 | 1621 | 0.2534 |
| SO03 | Sobral | -40.18 | -3.64 | 42729 | 17771 | 24958 | 0.5841 | 6397 | 1293 | 0.202126 |
| SO04 | Sobral | -40.18 | -3.64 | 42729 | 18203 | 24526 | 0.574 | 6397 | 1077 | 0.16836 |
| SO05 | Sobral | -40.18 | -3.64 | 42729 | 18166 | 24563 | 0.5749 | 6397 | 1078 | 0.168516 |
| SO06 | Sobral | -40.18 | -3.64 | 42729 | 18025 | 24704 | 0.5782 | 6397 | 1175 | 0.18368 |
| SO07 | Sobral | -40.18 | -3.64 | 42729 | 18266 | 24463 | 0.5725 | 6397 | 1038 | 0.162264 |
| SO08 | Sobral | -40.18 | -3.64 | 42729 | 17778 | 24951 | 0.5839 | 6397 | 1372 | 0.214476 |
| SO09 | Sobral | -40.18 | -3.64 | 42729 | 17109 | 25620 | 0.5996 | 6397 | 1614 | 0.252306 |
| SO10 | Sobral | -40.18 | -3.64 | 42729 | 18144 | 24585 | 0.5754 | 6397 | 1121 | 0.175238 |
| SQ201 | Santa Quitéria | -40.19 | -4.44 | 42729 | 21926 | 20803 | 0.4869 | 6397 | 931 | 0.145537 |
| SQ202 | Santa Quitéria | -40.19 | -4.44 | 42729 | 20474 | 22255 | 0.5208 | 6397 | 1209 | 0.188995 |
| SQ203 | Santa Quitéria | -40.19 | -4.44 | 42729 | 22355 | 20374 | 0.4768 | 6397 | 756 | 0.11818 |
| SQ204 | Santa Quitéria | -40.19 | -4.44 | 42729 | 21694 | 21035 | 0.4923 | 6397 | 924 | 0.144443 |
| SQ205 | Santa Quitéria | -40.19 | -4.44 | 42729 | 21306 | 21423 | 0.5014 | 6397 | 995 | 0.155542 |
| SQ206 | Santa Quitéria | -40.19 | -4.44 | 42729 | 21414 | 21315 | 0.4988 | 6397 | 1076 | 0.168204 |
| SQ207 | Santa Quitéria | -40.19 | -4.44 | 42729 | 22100 | 20629 | 0.4828 | 6397 | 854 | 0.1335 |
| SQ208 | Santa Quitéria | -40.19 | -4.44 | 42729 | 21255 | 21474 | 0.5026 | 6397 | 1100 | 0.171956 |
| SQ209 | Santa Quitéria | -40.19 | -4.44 | 42729 | 21990 | 20739 | 0.4854 | 6397 | 904 | 0.141316 |
| SQ210 | Santa Quitéria | -40.19 | -4.44 | 42729 | 22128 | 20601 | 0.4821 | 6397 | 873 | 0.13647 |
| JM01 | Jaguaretama | -38.74 | -5.57 | 42729 | 20277 | 22452 | 0.5255 | 6397 | 760 | 0.118806 |
| JM02 | Jaguaretama | -38.74 | -5.57 | 42729 | 16562 | 26167 | 0.6124 | 6397 | 2257 | 0.352822 |
| JM03 | Jaguaretama | -38.74 | -5.57 | 42729 | 20399 | 22330 | 0.5226 | 6397 | 844 | 0.131937 |
| JM04 | Jaguaretama | -38.74 | -5.57 | 42729 | 20009 | 22720 | 0.5317 | 6397 | 974 | 0.152259 |
| JM05 | Jaguaretama | -38.74 | -5.57 | 42729 | 19541 | 23188 | 0.5427 | 6397 | 1137 | 0.17774 |
| JM06 | Jaguaretama | -38.74 | -5.57 | 42729 | 20199 | 22530 | 0.5273 | 6397 | 875 | 0.136783 |
| JM07 | Jaguaretama | -38.74 | -5.57 | 42729 | 20035 | 22694 | 0.5311 | 6397 | 947 | 0.148038 |
| JM08 | Jaguaretama | -38.74 | -5.57 | 42729 | 19902 | 22827 | 0.5342 | 6397 | 934 | 0.146006 |
| JM09 | Jaguaretama | -38.74 | -5.57 | 42729 | 18218 | 24511 | 0.5736 | 6397 | 1371 | 0.214319 |
| JM10 | Jaguaretama | -38.74 | -5.57 | 42729 | 20362 | 22367 | 0.5235 | 6397 | 808 | 0.126309 |
| JG201 | Jaguaribe | -38.65 | -5.93 | 42729 | 13965 | 28764 | 0.6732 | 6397 | 3016 | 0.471471 |
| JG202 | Jaguaribe | -38.65 | -5.93 | 42729 | 17086 | 25643 | 0.6001 | 6397 | 1777 | 0.277786 |
| JG203 | Jaguaribe | -38.65 | -5.93 | 42729 | 18433 | 24296 | 0.5686 | 6397 | 1076 | 0.168204 |
| JG204 | Jaguaribe | -38.65 | -5.93 | 42729 | 17808 | 24921 | 0.5832 | 6397 | 1335 | 0.208692 |
| JG205 | Jaguaribe | -38.65 | -5.93 | 42729 | 17921 | 24808 | 0.5806 | 6397 | 1184 | 0.185087 |
| JG206 | Jaguaribe | -38.65 | -5.93 | 42729 | 18504 | 24225 | 0.5669 | 6397 | 1076 | 0.168204 |
| JG207 | Jaguaribe | -38.65 | -5.93 | 42729 | 17777 | 24952 | 0.584 | 6397 | 1335 | 0.208692 |
| JG208 | Jaguaribe | -38.65 | -5.93 | 42729 | 18630 | 24099 | 0.564 | 6397 | 982 | 0.153509 |
| JG209 | Jaguaribe | -38.65 | -5.93 | 42729 | 18239 | 24490 | 0.5731 | 6397 | 1113 | 0.173988 |
| JG210 | Jaguaribe | -38.65 | -5.93 | 42729 | 18510 | 24219 | 0.5668 | 6397 | 1070 | 0.167266 |
| MN01 | Morada Nova | -38.51 | -5.32 | 42729 | 19827 | 22902 | 0.536 | 6397 | 1610 | 0.25168 |
| MN02 | Morada Nova | -38.51 | -5.32 | 42729 | 22561 | 20168 | 0.472 | 6397 | 892 | 0.13944 |
| MN03 | Morada Nova | -38.51 | -5.32 | 42729 | 22474 | 20255 | 0.474 | 6397 | 909 | 0.142098 |
| MN04 | Morada Nova | -38.51 | -5.32 | 42729 | 22343 | 20386 | 0.4771 | 6397 | 944 | 0.147569 |
| MN05 | Morada Nova | -38.51 | -5.32 | 42729 | 22806 | 19923 | 0.4663 | 6397 | 836 | 0.130686 |
| MN06 | Morada Nova | -38.51 | -5.32 | 42729 | 22970 | 19759 | 0.4624 | 6397 | 782 | 0.122245 |
| MN07 | Morada Nova | -38.51 | -5.32 | 42729 | 21511 | 21218 | 0.4966 | 6397 | 1183 | 0.18493 |
| MN08 | Morada Nova | -38.51 | -5.32 | 42729 | 21378 | 21351 | 0.4997 | 6397 | 1236 | 0.193216 |
| MN09 | Morada Nova | -38.51 | -5.32 | 42729 | 22869 | 19860 | 0.4648 | 6397 | 783 | 0.122401 |
| MN10 | Morada Nova | -38.51 | -5.32 | 42729 | 22298 | 20431 | 0.4782 | 6397 | 887 | 0.138659 |

Table S2. Relatedness of each individual relationship available at: https://datadryad.org/stash/share/decFj9rWvlJhEjEmmc8hJkrAWF1B7hOAI7osLX-JV70

Table S3. Value of marginal Likelihood for each cluster from FastStructure**.**

| **Value of clusters** | **Value of marginal Likelihood** |
| --- | --- |
| **K1** | -0.673223 |
| **K2** | -0.685993 |
| **K3** | -0.679993 |
| **K4** | -0.686121 |
| **K5** | -0.677497 |
| **K6** | -0.676581 |
| **K7** | -0.673295 |
